# Noninvasive assessment of fluid responsiveness for emergency abdominal surgery in dogs with pulmonary hypertension: Insights into high-risk companion animal anesthesia

**DOI:** 10.1371/journal.pone.0241234

**Published:** 2020-10-23

**Authors:** Kazumasu Sasaki, Shuzo Yamamoto, Tatsushi Mutoh

**Affiliations:** 1 Small Animal Emergency and Critical Care Service, Sendai Animal Care and Research Center, Sendai, Japan; 2 Akita Cerebrospinal and Cardiovascular Center, Akita, Japan; 3 Institute of Development, Aging and Cancer, Tohoku University, Sendai, Japan; Scuola Superiore Sant'Anna, ITALY

## Abstract

**Objective:**

Optimizing cardiac stroke volume during high-risk surgical anesthesia is of particular interest with regard to a therapeutic target to reduce the incidence of postoperative complications. However, intensive fluid management in critically ill small animals with pulmonary hypertension (PH) has been empirically performed, and thus it can be challenging. Stroke volume variation (SVV) has been used as a dynamic preload predictor of fluid responsiveness. We hypothesized that if SVV exhibited robust reliability in the setting of hemodynamically unstable condition, it would provide more precise information on fluid resuscitation to translate it into veterinary anesthesia. Thus the aim of this study was to investigate the utility of SVV measured by the electrical velocimetry (EV) method for predicting fluid responsiveness in dogs with PH.

**Methods:**

Sixteen dogs undergoing emergency abdominal surgery and diagnosed with PH secondary to myxomatous mitral valve disease (MMVD) on preoperative transthoracic echocardiogram were included. Dogs were randomly assigned to 2 groups with and without inotropic cardiac support with dobutamine. Hemodynamic measurements including stroke volume and SVV derived from the EV device were performed under general anesthesia before (baseline) and after surgery (fluid challenge with a colloid solution defined by a SV increase of ≥ 10%).

**Results:**

In both groups, SVV elevated significantly after abdominal surgery compared with baseline. In dobutamine infused group, the SVV values decreased significantly after fluid challenge (*P* < 0.05) with a greater number of responders than saline infused control group (*P* < 0.01). Receiver operating curve analysis of SVV confirmed high positive predictive value for dogs during dobutamine infusion (*P* < 0.05; cut-off value of 15%; specificity 90%, sensitivity 82%).

**Conclusions:**

Noninvasive EV monitoring may be useful for the prediction of fluid responsiveness in critically ill dogs with left-sided heart failure-related PH. This normalization of dynamic preload indices, which could be achieved more precisely under inotropic support, may prevent further detrimental consequence of fluid loading.

## Introduction

Optimizing cardiac stroke volume during surgical anesthesia is of particular interest with regard to a therapeutic target to reduce the incidence of postoperative complications. Pulmonary hypertension (PH) is related to the risk for development and severity of clinically significant congestive heart failure [1]. There is now growing appreciation that left-sided heart failure is known to cause PH in elderly patients and is associated with increased left-sided filling pressure that may lead to pulmonary venous hypertension and post-capillary PH [2]. Many cases of PH in animals are related to left side heart disease. Myxomatous mitral valve disease (MMVD) is a very common degenerative disease seen in dogs, particularly older small breed dogs, and a diagnostic criteria of PH has also been proposed in veterinary practice [3]. However, the principles of fluid therapy and how they influence critical decisions on intensive care management have been underestimated in the field of veterinary anesthesia despite increased population of elderly companion animals with congestive heart failure [4].

Assessment of hemodynamic and volume status can be achieved by intensive fluid management guided by a combination of cardiac output (CO) monitoring and preload indices. Pulmonary or transpulmonary thermodilution and uncalibrated arterial pressure waveform analysis can provide valuable information for understanding hemodynamically unstable conditions in experimental animals [5–15]. However, most of the advanced monitoring systems are neither simple nor easy to use in current veterinary practice because of their invasiveness and/or complexity (e.g., arterial cannulation in large vessel and related risks for infection and bleeding from the puncture sites), particularly their practical applications to client-owned animals.

Recently, a new noninvasive monitoring system, without the need for external calibration, using the electrical velocimetry (EV) method has been introduced in dogs with experimental cardiac surgery [16–19]. In addition to CO and stroke volume (SV) measurements, the EV also displays stroke volume variation (SVV) automatically in real-time [17, 19]. Several lines of evidence suggest that SVV is better than the static methods for determining preload, such as central venous pressure and pulmonary artery occlusion pressure, for predicting fluid responsiveness and guiding fluid therapy in critically ill patients [9, 20] and dogs undergoing experimental cardiac surgery [16–18, 21]. Despite its potential for practical acceptance in terms of CO measurements and ease of use, limited clinical data are available regarding the role of noninvasive SVV assessment as a guide to intensive fluid management during high-risk surgical anesthesia in dogs.

We hypothesized that if SVV exhibited robust reliability in “*real-world*” emergency surgical setting of hemodynamically unstable condition, it would provide more precise information on fluid resuscitation to translate it into veterinary anesthesia and critical care. Thus the aim of this study was to investigate the utility of EV-based SVV for predicting fluid responsiveness in dogs with PH secondary to MMVD undergoing emergency abdominal surgery. In this study, fluid challenge was performed in the presence of inotropic support with dobutamine to ameliorate the ‘false-positive’ effect of SVV elevation attributable to PH in lowering afterload [22, 23].

## Materials and methods

This prospective, randomized study was carried out at the Small Animal Emergency and Critical Care Service, Sendai Animal Care and Research Center. The owners of the patients provided written informed client consent before enrolment for participation in this experiment. The study protocol was reviewed and approved by the Institutional Ethical Committee on Animal Use of Akita Cerebrospinal and Cardiovascular Center (18–02).

### Animals

Client-owned dogs receiving emergency abdominal surgery and diagnosed with PH secondary to myxomatous mitral valve disease (MMVD) stage B2 by the Consensus Statements of the American College of Veterinary Internal Medicine [3, 24] based on preoperative exams (i.e., physical examination, complete blood cell count and serum biochemistry profile, and transthoracic echocardiogram) were included. All dogs were first-time patients, and the patient’s owner was not aware of the presence of mitral valve disease. Although increased respiratory effort, tachypnea, ascites, and hepatomegaly were confirmed in some dogs, all dogs were negative for blood microfilariae and the adult heartworm antigen test (CHW Ag test kit; Kyokuto Pharmaceutical Co. Ltd., Japan). We conducted conventional examinations using ultrasound units (Canon Xario 100; Canon Medical Systems, Japan) equipped with sector (2.8−6.2 MHz), convex (3.3−11 MHz), and linear (5−18 MHz) transducers. The ratio of left atrial diameter to aortic root diameter (LA/Ao) was obtained in the right parasternal short-axis 2D images of the heart base [25]. Using M-mode, left ventricular end-diastolic diameter (LVEDD) in the diastolic period was measured on a right parasternal short-axis view at the papillary muscle level [26]. LVEDD was normalized for body weight (BW) as follows: LVEDD/Bw^0.294^ [27]. Left ventricular systolic function was assessed by fractional shortening (FS), which was measured by M-mode. The peak tricuspid value (TRV) was measured using continuous-wave Doppler imaging on a left apical 4-chamber view [28]. Dogs were deemed to have PH if TRV > 3 m/s [28]. Risk for general anesthesia of animals to tolerate emergency surgical intervention has been carefully assessed by veterinary anesthesiologists (KS and TM). Animals were randomly assigned to 2 groups with and without inotropic support of dobutamine infusion (**S1 Fig**). Randomization was generated using a computer software (http://www.randomization.com/) and their treatment allocations were established by sealed manila envelopes [29].

### Anesthesia protocol and instrumentation

The details of the experimental setup have been fully described elsewhere [16]. A 22 gauge (G) over-the-needle catheter (Insyte-W; Becton, Dickinson & Co., UT, USA) was placed in a cephalic vein and lactated Ringer’s solution (Lactec; Otsuka Pharmaceutical Factory, Inc., Japan) was infused intravenously (IV) at 5 ml kg^-1^ hour^-1^. Following preoxygenation with 100% oxygen (4 L minute^-1^), dogs were premedicated with an IV injection of midazolam (0.2 mg kg^−1^; Midazolam; Taiyo Teva Seiyaku, Japan) and fentanyl (10 μg kg^−1^; Fentanyl; Daiichi Sankyo Propharma Co. Ltd., Japan). Anesthesia was induced with 5% sevoflurane (Sevofrane; Maruishi Pharmaceutical Co. Ltd., Japan) delivered by facemask [30–35]. After endotracheal intubation, anesthesia was maintained with sevoflurane delivered in an oxygen: air mixture at a flow rate of 2 L minute^-1^ with an inspired fraction of oxygen (FIO_2_) of 0.6 via a semi-closed rebreathing circle anesthetic system. Continuous side-stream gas sampling was used to measure end-tidal carbon dioxide (Pe´CO_2_), end-tidal sevoflurane concentration (Fe´Sevo via a straight connector attached between the Y-piece of the circuit and the endotracheal tube. Monitoring including FIO_2_, FE´ Sevo, Pe´CO_2_, and oxygen saturation of haemoglobin (SpO_2_) were analyzed by using a biological information monitoring device (Life Scope BSM-5192; Nihon Kohden Corp., Japan) with a built-in automatic calibration system. Mechanical ventilation was instituted using a ventilator (PRO-NEXT +i/+s; ACOMA Medical Industry Co., Tokyo, Japan) immediately following the induction of anesthesia using an A/C mode [initial setting: volume-controlled ventilation with tidal volume (V_T_) of 10 ml kg^-1^, respiratory rate (*f*_R_) of 10–16 breaths minute^−1^ and FIO_2_ of 0.6 adjusted to maintain eucapnia of Pe´CO_2_ 33–45 mmHg, 4.4–5.9 kP]. Rectal temperature was maintained at 37–38°C using a forced-air patient warmer (Bair Hugger; 3M Japan Ltd., Tokyo, Japan). Anesthesia was maintained with sevoflurane (1.0–2.0%) in 60% oxygen for an adequate depth of anesthesia as described previously [16]. Intraoperative analgesia was provided by a constant rate infusion (CRI) of remifentanil (0.1–0.3 μg/kg/min) (Ultiva; Janssen Pharmaceutical K.K, Japan).

Mean arterial pressure (MAP) was measured via a 20 G catheter, 30 mm catheter (BD Insyte-A; Becton, Dickinson & Co.) placed in the right dorsal pedal artery using a securement device [36] and connected to the pressure transducer (DX-360; Nihon Kohden, Tokyo, Japan). A 19G, 30.5 cm central venous catheter (Intracath, Becton Dickinson Infusion Therapy Systems) was placed in the jugular vein with the tip close to the right atrium, with positioning based on observation of characteristic pressure waves of central venous pressure (CVP).

A noninvasive EV system (Aesculon; Osypka Medical, Irvine, CA, USA) was established by placing four electrocardiographic electrodes for continuous measurements of heart rate (HR), SV, CO, and SVV (*See* **S2 Fig**). Briefly, two electrodes were placed at the left side of the neck adjacent to the common carotid artery, and two electrodes were placed on the left lower thorax [16]. The EV method uses thoracic electrical bioimpedance, which relates changes in aortic blood flow and blood volume to interpret the ohmic equivalent mean aortic flow acceleration and HR correction and calculated SV and CO using the Bernstein-Osypka equation [37]. Impedance and electrocardiogram data were displayed on the bioimpedance monitor and the impedance SV was recorded automatically in an internal database at 30-second intervals. The values of CO and SV were indexed to body surface area and body weight (CI and SVI, respectively). SVV was determined automatically using the equation (S_Vmaximum −_ SV_minimum_)/SV_mean_ over 10 respiratory cycles [17].

### Study protocol

This study measured hemodynamic variables in sevoflurane-anesthetized dogs at Fe´Sevo 2.36% [1.0 minimum alveolar concentration (MAC)] [5] with a constant rate infusion of remifentanil (0.05 μg/kg/min) under mechanical ventilation. Animals were allowed to stabilize for 15 min to receive either dobutamine (Dobutrex; Kyowa Pharmaceutical Industry Co., Ltd., Osaka, Japan) (10 μg kg^-1^ min^-1^) or vehicle sodium chloride 0.9% w/v (Normal saline; Otsuka Pharmaceutical Co., Ltd., Tokyo, Japan) at a constant infusion rate (2 ml kg^-1^ hour^-1^) [38, 39].

**Fig 1:**
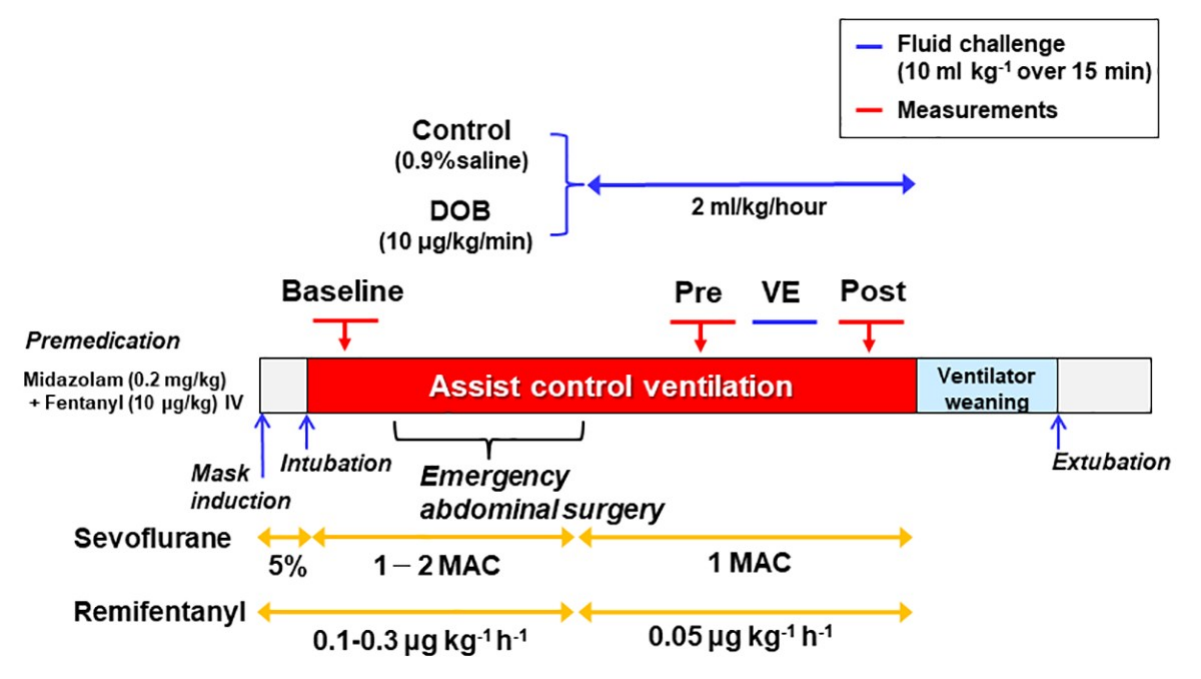
Study design. Experimental groups (Control and DOB) and protocol of EV monitoring during three different volumetric conditions [Baseline and before (Pre) and after (Post) volume expansion (VE)].

Hemodynamic measurements were performed during three different conditions: 1) Normovolemia (preoperative baseline measurements); 2) Hypovolemia (initial measurements after abdominal wall closure); and 3) Volume expansion [measurements after fluid loading (10 ml kg^-1^ IV over 15 minutes) of a colloid solution (Saviosol, 3% Dextran-40; Otsuka Pharmaceutical, Tokyo, Japan)]. Dogs were considered fluid responsive if SVI measured immediately after completion of the fluid challenge increased by ≥10% [40, 41] (**Fig 1**). Measurements of each volumetric stage were obtained during a 15 min recording after a short period of stabilization. After completion of the fluid challenges, the dogs were allowed to recover from anesthesia with appropriate postoperative analgesia and intensive care in the clinic (**S2 Fig**).

### Statistical analysis

The main outcomes evaluated in this study were SVI and SVV in response to fluid challenge at each time point. Power analysis revealed a sample size of seven dogs to detect a 40% effect in SVV (standardized difference of 1.5: target difference/standard deviation [SD]) for a level of significance of 0.05 and a power of 80% to be achieved [42].

All variables were compared to the baseline (values before each intervention) and among the groups (DOB vs. Control). Data are expressed as mean ± SD, unless otherwise mentioned. Normally distributed data among groups were determined with analysis of variance (ANOVA), and non-normally distributed variables were analyzed with Kruskal-Wallis test. When statistical significance level has been reached, parametric (Bonferroni test) and non-parametric (Scheffe test) univariate comparisons were performed. Fisher’s exact test was used to compare the categorical variables. The predictive ability of a variable for fluid responsiveness was assessed using a receiver operating characteristic (ROC) curve calculated with a 95% confidence interval. For each variable, a threshold value was determined to maximize both sensitivity and specificity. A *P*-value of 0.05 was considered statistically significant. Statistical analyses were performed using SPSS (version 24.0; IBM, IL, USA), Prism (version 8: GraphPad Software, CA, USA), and SigmaPlot (version 13.0; Systat Software, Inc., CA, USA).

## Results

Baseline characteristics of the 16 dogs underwent emergency abdominal surgery (n = 14 ovariohysterectomy for pyometra; n = 2 cholecystectomy for ruptured gall bladder) are presented in **Table 1**. All dogs were extubated and recovered without procedure- or therapy-related complications or death.

**Table 1:** Baseline characteristics.

	Overall (n = 16)	Control (n = 8)	DOB (n = 8)
Age (years)	11 ± 4 (6–15)	11 ± 4 (6–15)	12 ± 3 (8–15)
Body weight (kg)	12 ± 4 (7–20)	12 ± 4 (7–20)	15 ± 6 (8–25)
Anesthesia time (min)	134 ± 13 (113–155)	134 ± 13 (113–155)	132 ± 9 (122–149)
Operation time (min)	67 ± 21 (48–105)	67 ± 21 (48–105)	61 ± 17 (43–87)
Intraoperative weight loss (g)^1)^	340 ± 107 (230–520)	340 ± 107 (230–520)	397 ± 161 (213–625)
Total volume of IV fluids (mL)	166 ± 19 (154–201)	163 ± 20 (154–197)	169 ± 19 (144–201)
*Clinical symptoms*:			
Cardiac murmur (grade)*	4 (3–5)	4 (3–5)	4 (3–5)
Increased respiratory effort (n)	4	2	2
Tachypnea (n)	7	3	4
Ascites (n)	6	3	3
Hepatomegaly (n)	9	5	4
*Echocardiographic indices*:			
LA/Ao	2.29 ± 0.44 (1.77–3.18)	2.27 ± 0.42 (1.87–3.18)	2.31 ± 0.48 (1.77–3.01)
LVEDDn	2.08 ± 0.49 (1.27–2.91)	2.11 ± 0.52 (1.27–2.81)	2.06 ± 0.49 (1.58–2.91)
FS (%)	45.33 ± 6.48 (32.81–53.29)	45.05 ± 7.31 (35.12–53.29)	45.62 ± 6.03 (32.81–51.11)
Peak TRV (m/s)	3.78 ± 0.21 (3.57–4.24)	3.75 ± 0.22 (3.57–4.18)	3.82 ± 0.21 (3.57–4.24)

Data are expressed as mean ± SD (range).

*Data are expressed as median (range).

DOB, dobutamine; LA/Ao, left atrial to aortic root ratio; LVEDDn, normalized left-ventricle end-diastolic diameter indexed; FS, fractional shortening; TRV, tricuspid regurgitation velocity; IV, intravenous.

^1)^Estimated weight counted by blood-soaked gauze and resected tissue.

**Fig 2:**
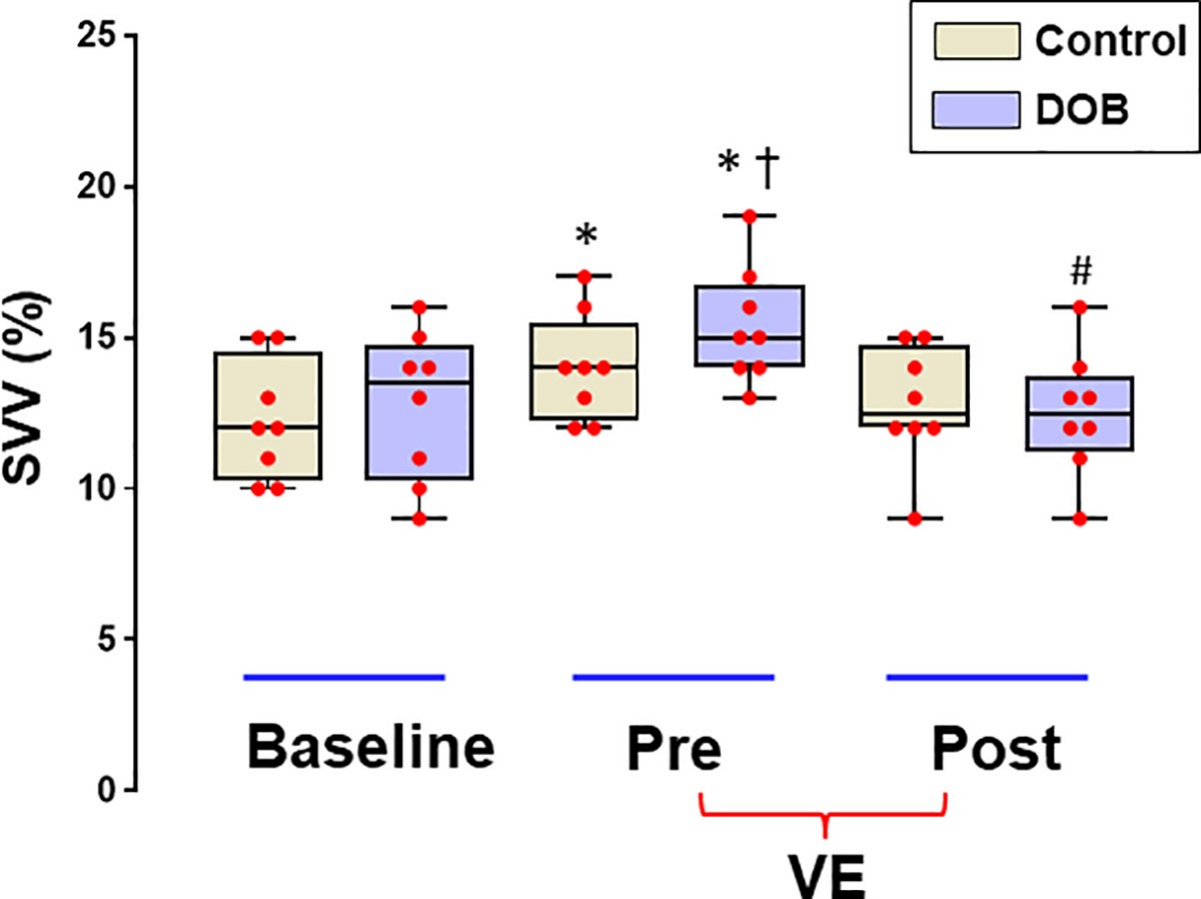
Box-and-whisker plots of SVV during 3 different volumetric conditions. VE, volume expansion; DOB, dobutamine. *Significantly different from Baseline within each group (*P* < 0.05). ^#^Significantly different from the values before VE (Pre-VE) within each group (*P* < 0.05). ^†^Significantly different from Control (*P* < 0.05).

Hemodynamic data with and without DOB groups under different volumetric conditions are shown in **Table 2**. There were no statistically significant group differences between the baseline phase. Hypovolemia induced by intraoperative bleeding and resected tissue (368 ± 135 g) (**Table 1**) caused a significant increase of SVV as compared to baseline (*P* = 0.003; within subject effect; two-way ANOVA repeated measures), the degree which was greater in the DOB group (*P* = 0.04; between subject effect) (**Fig 2**).

**Table 2:** Hemodynamic variables under different volumetric conditions.

	Baseline		Pre-VE		Post-VE	
	Control	DOB	Control	DOB	Control	DOB
HR (beats min^−1^)	101 ± 11	102 ± 10	109 ± 10	111 ± 14	112 ± 13	114 ± 11
MAP (mmHg)	84 ± 6	87 ± 6	89 ± 4	94 ± 3	91 ± 4	95 ± 4
CVP (mmHg)	6 ± 5	6 ± 4	6 ± 3	5 ± 4	7 ± 4	7 ± 4
SVI (mL beat^−1^ kg^−1^)	1.55 ± 0.20	1.58 ± 0.17	1.54 ± 0.20	1.61 ± 0.18*^†^	1.62 ± 0.22	1.80 ± 0.27^#^^†^
CI (L min^−1^ m^−2^)	2.6 ± 0.5	2.7 ± 0.5	2.7 ± 0.3	3.0 ± 0.6*^†^	3.0 ± 0.7	3.5 ± 0.7^#^^†^
SVV (%)	12.1 ± 2.1	12.4 ± 2.5	14.1 ± 2.4*	15.6 ± 2.0*^†^	12.4 ± 1.9	12.0 ± 1.6^#^

VE, volume expansion; DOB, dobutamine; HR, heart rate; MAP, mean arterial pressure; CVP, central venous pressure; SVI, stroke volume index; CI, cardiac index; SVV, stroke volume index.

*Significantly different from Baseline measured before starting surgery within each group (*P* < 0.05).

^#^Significantly different from the values before VE (Pre-VE) within each group (*P* < 0.05).

^†^Significantly different from Control (*P* < 0.05).

No significant differences were observed in the effect of volume expansion in control group (*P* = 0.229; Pre-VE vs. Post-VE).

**Fig 3:**
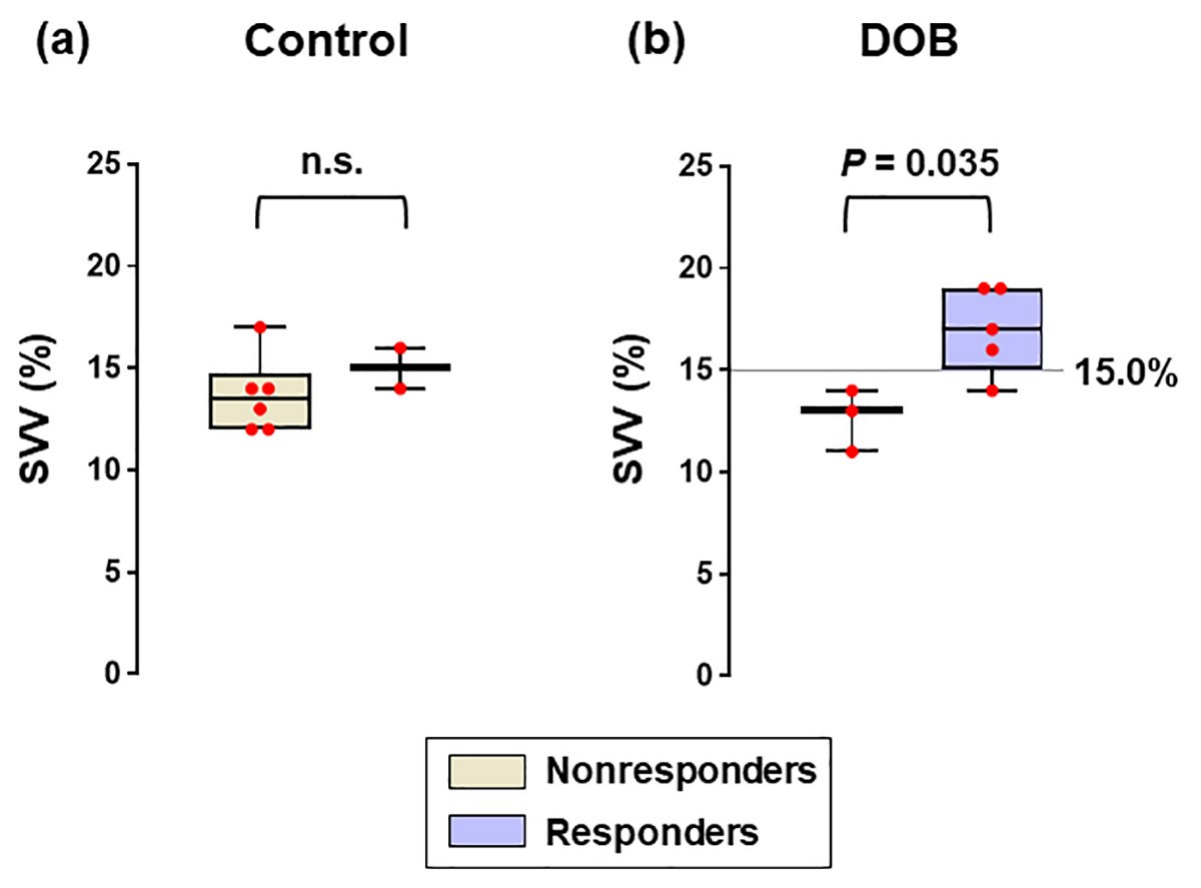
Box-and-whisker plots of SVV before volume expansion. The straight line depicts the SVV cut-off to discriminate fluid responders of SVI ≥ 10%. Data are expressed as median values and interquartile ranges with scatter plots. n.s., not significant; DOB, dobutamine.

In DOB groups, SVV decreased significantly after volume expansion (*P* < 0.05), in which dobutamine infused group had a greater number of responders (63%) than saline infused group (25%) (*P* < 0.001). Before volume loading, the values of SVV were significantly higher in responders defined by an increase of SVI ≥ 10% (*P* = 0.035), whereas those of SVV were not (*P* = 0.39) (**Fig 3**).

**Fig 4:**
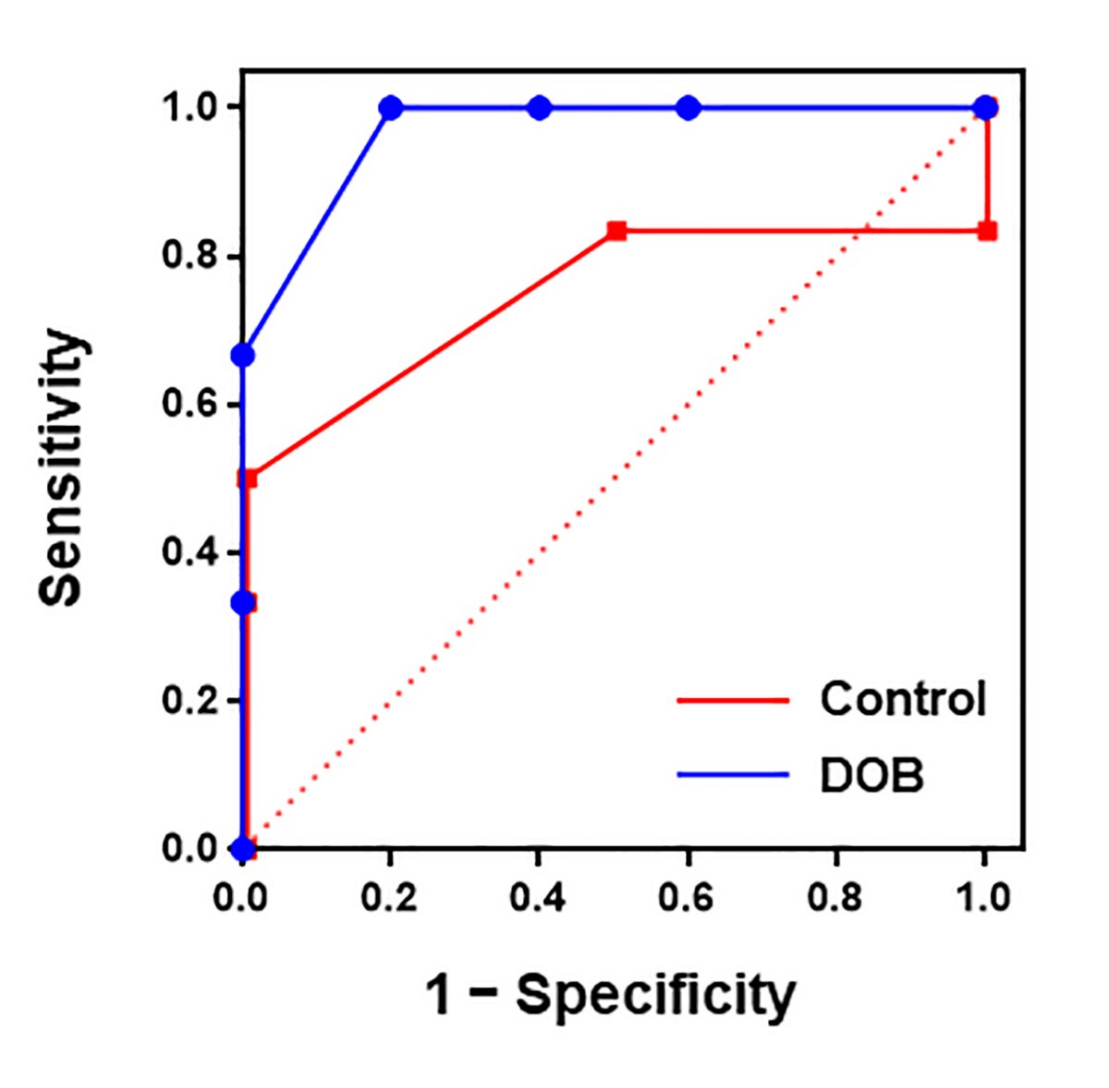
Prediction of fluid responsiveness by the ROC curves of SVV. The 45-degree diagonal line indicates the reference line of no-discrimination. DOB, dobutamine.

The ROC curve analyses for SVV illustrated that fluid responsiveness for the identification of responders was predicted better with DOB infused group [AUC = 0.97 ± 0.06 (standard error), *P* = 0.037] than with saline control group (AUC = 0.75 ± 0.18, *P* = 0.32) (**Fig 4**). The optimal cut-off values were 15.0% for SVV during DOB infusion (90% specificity and 82% sensitivity) (**Table 3**). Corresponding grey zone limits of SVV were between 13.5% and 16.5%.

**Table 3:** Prediction of fluid responsiveness.

	Cut-off value	AUC (95%CI)	*P*-value	Specificity (%)	Sensitivity (%)	Grey zone
**Control (n = 8)**	N/A	0.79 (0.47–1.11)	0.243	N/A	N/A	N/A
**DOB (n = 8)**	15.0%	0.97 (0.84–1.09)	0.037	90	82	13.5–16.5%

AUC, area under the receiver operating characteristics (ROC) curve; CI, confidence interval; DOB, dobutamine; Grey zone, range of values with a sensitivity <90% or specificity <90%; N/A, not available.

## Discussion

The principal finding of this study is that noninvasive SVV analyzed by the EV device is valuable for the prediction of fluid responsiveness in dogs with PH and MMVD undergoing emergency abdominal surgery. This normalization of dynamic preload indices, which could be achieved more precisely under inotropic support, may prevent further detrimental consequence of fluid loading.

In this study, the EV-derived SVV is assumed to be an adequate predictor of fluid responsiveness in the setting of PH secondary to MMVD. However, it should be noted here that preload requirements for PH differ substantially based on whether afterload is normal or increased. The PH and volume loading can induce displacement of the interventricular septum toward the LV and subsequent decline of RV perfusion. In this setting, intravascular volume dehydration should be needed to avoid exacerbating bi-ventricular cardiac dysfunction because of less chance of improvement on RV contractility over a wide range of filling pressures due to flatter Starling curve of RV than the LV [43]. This may lead to RV global dysfunction and secondarily with LV systolic dysfunction, making the animals none or less responsive to fluid loading [44], as demonstrated in the Control group.

In this regard, our results suggest that supplementary inotropic support improved fluid responsiveness to dogs with MMVD-related PH. Dobutamine is an inotrope that acts via β_1_ receptor stimulation, but it may also cause vasodilatation due to β_2_ effects with increasing doses [45]. In this study we used dobutamine at low dose (5–10 μg kg^-1^ min^-1^) because it restores pulmonary artery/RV coupling in a canine study [46] and improves myocardial contractility and pulmonary vascular resistance in patients with left heart failure [47]. The beneficial role of low-dose dobutamine in improving hemodynamics in patients after RV infarction has also been demonstrated [48]. Although higher dose (20 μg kg^-1^ min^-1^) should be avoided in this setting because of the risk of β_2_-mediated vasodilatation and hypotension in healthy dogs [39]. Sinus tachycardia and tachyarrhythmia should also be considered as a risk for high-dose dobutamine despite an inadequate oxygen delivery despite the correction of abnormalities in RV preload, afterload, and ischemia, resulting in a poor prognostic indicator in PH [49]. In fact, we usually apply inotropic therapy with dobutamine below 15 μg kg^-1^ min^-1^ to maintain CO close to normal range by minimizing adverse drug effects in emergency surgical patients with variety range of systemically disease histories [10, 11, 14].

Although only a few studies have directly compared SVV with other estimates of dynamic indices [50, 51], our present and preliminary data demonstrated that both pulse pressure variation (PPV) and systolic pressure variation (SPV) showed comparable performance in predicting fluid responsiveness to raise the SVI and CI in dogs receiving high-risk veterinary anesthesia [21]. The optimal threshold value for SVV to discriminate between responders and nonresponders in dogs anesthetized in a variety of clinical scenarios is still limited because many factors (e.g., type and depth of anesthesia, ventilation modes, heart-lung interaction, and comorbid cardiac failure, etc.) may affect interpretation of hypovolemia [52]. Previous studies have shown that SVV threshold values of 13.5–15% for a 10% SVI response in dogs undergoing cardiac surgery (73–83% sensitivity and 60–84% specificity) [16, 17] are in agreement with the present study of dogs with PH receiving the inotropic support. These findings support the feasibility of EV at least in part, for assessing intraoperative fluid responsiveness in anesthetized mechanically ventilated dogs such as continuous/synchronized intermittent mandatory ventilation [18, 21].

This study has some limitations that may concern its readers. Currently, various functional haemodynamic variables (e.g., SVV, PPV, SPV, or plethysmography variability index) have been employed to estimate volume responsiveness in dogs [16–18, 51, 53–59]. Although this is the initial canine study using the EV device for intraoperative fluid management in dogs with PH, it is still limited by its relatively small sample size. Because of the mini-fluid challenge protocol (approximately 166 mL), given for a short period over 15 minutes, changes of clinical non-cardiac fluid parameters such as fluid balance, urine output, tachycardia, and skin turgor were not studied. In addition, our results are limited by the small and varying number of fluid response estimates obtained for dogs under a specific emergency condition. Our findings might thus not be directly applicable to other situations or to human patients other than the population enrolled. Nevertheless, the strengths of this study include its prospective nature and its measurement of SVV and CO using the *noninvasive* EV methodology in dogs under intravascular hypovolemia attributable to losses of body fluids to 'third spaces' (e.g., pyometra, ascites, pleural effusions, interstitial edema). Furthermore, this study was carried out before and after abdominal wall closure for ethical aspects of veterinary patients’ safety. While previous studies have suggested that dynamic preload indicators such as PPV and SVV are able to predict fluid responsiveness during major abdominal surgery [60, 61], there are still some controversies regarding the predictive values of fluid responsiveness compared to values obtained in the ICU setting [44]. Future studies are expected to establish the utility of EV-based goal-directed therapy in larger populations to ensure adequate hemodynamic stabilities throughout the phase of surgical anesthesia and improved outcomes of the emergency canine patients.

## Conclusions

Although no standardized criteria exist to define hemodynamic consequences in canine high-risk surgical patients under PH secondary to MMVD, the results in this study using a noninvasive EV technique for monitoring both dynamic preload status and CO suggest the technique would be valuable for estimating fluid responsiveness.

## Supporting information

S1 Fig Flow diagram of the study selection process. (TIF)

S2 Fig Schemas of intensive monitoring at the Sendai Animal Care and Research Center (SACRC). (a) Anesthesia monitor and electrical velocimetry (EV) device, (b) Automated anaesthesia record system, (c) Infusion pumps for controlled IV fluid administration, and (d) Positions of electrodes for noninvasive EV monitoring in the clinical setting of a dog suffering from septic shock due to ruptured gallbladder. In our institute, hemodynamically unstable small animal patients, the EV system is routinely used for monitoring of surgical anesthesia.
(TIF)
