# Supplementary figures and images for: Noninvasive assessment of fluid responsiveness for emergency abdominal surgery in dogs with pulmonary hypertension: Insights into high-risk companion animal anesthesia
Source: PLoS One. 2020 Oct 23;15(10):e0241234. doi: 10.1371/journal.pone.0241234 (PMC7584187; doi:10.1371/journal.pone.0241234)

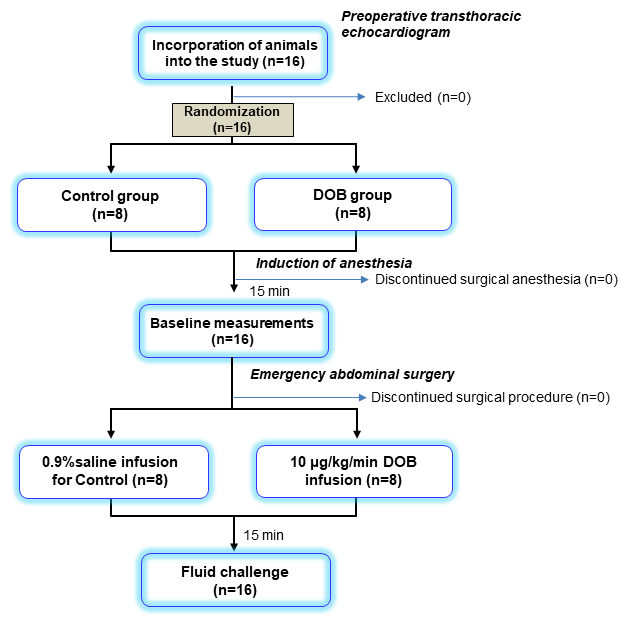

Supplement: S1 Fig — (TIF) [file pone.0241234.s001.tif]

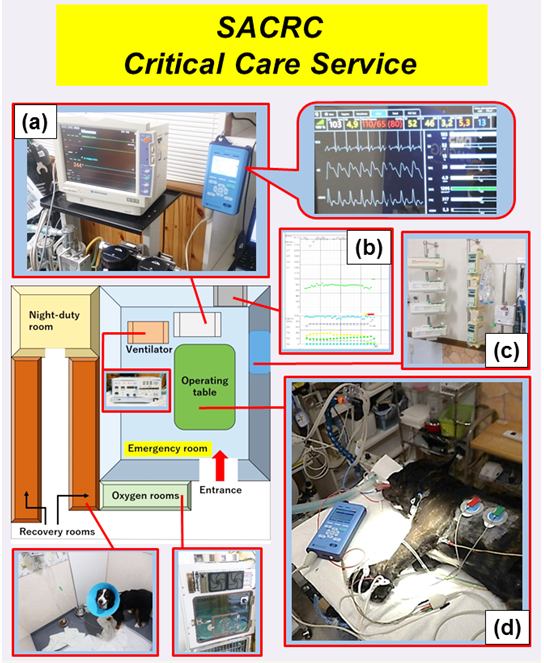

Supplement: S2 Fig — (a) Anesthesia monitor and electrical velocimetry (EV) device, (b) Automated anaesthesia record system, (c) Infusion pumps for controlled IV fluid administration, and (d) Positions of electrodes for noninvasive EV monitoring in the clinical setting of a dog suffering from septic shock due to ruptured gallbladder. In our institute, hemodynamically unstable small animal patients, the EV system is routinely used for monitoring of surgical anesthesia. (TIF) [file pone.0241234.s002.tif]
